# A Sketch of the Climate of the Valley of the Mississippi

**Published:** 1832

**Authors:** Daniel Drake


### WESTERN JOURNAL

OF THE

### MEDICAL AND PHYSICAL SCIENCES.

APRIL, MAY, AND JUNE, 1832.

### Essays and Cases.

ART. I.—*A Sketch of the Climate of the Valley of the Mississippi.*—By DANIEL DRAKE, M. D.

The vast extent of the Valley of the Mississippi, necessarily imparts to its climate a diversified character. The new states and territories already organized within its limits, are eight in number; while portions of two of the old states extend into the same region. The new may be divided into warm and temperate. The former are Louisiana, Mississippi, Alabama, in part, and the south half of the Territory of Arkansaw;—the latter, Tennessee, Kentucky, Ohio, in part, Indiana, Illinois, and Missouri. The first group lies between the latitudes of 30 deg. and 60 deg.; the second, extends from the paralell last mentioned, to that of 42 deg. Hence the first division corresponds with South Carolina and Georgia—the second, with North Carolina, Virginia, Maryland, Delaware, Jersey, and the southern part of New York.

In estimating the change of climate, to which emigrants from the old states will subject themselves, in settling in the new, it should be recollected, that Tennessee is in the rear of N. Carolina, and Kentucky of Virginia; and that Ohio, Indiana, Illinois, and Missouri, stretch out behind Pennsylvania, Maryland, Delaware, New Jersey, and the southern part of New York. The Newenglander, and Newyorker, north of the mountains of West Point, should bear in mind, that his emigration is not to the *west*, but *south-west;* and as necessarily brings him into a warmer climate, as when he seeks the shores of the Delaware, Potomac, or James' River.

Besides variations of latitude, several causes conspire to produce diversities in climate. Between the lower Mississippi states, and the portions of the old states corresponding with them in latitude, these causes are neither numerous nor powerful; and hence, on the same parallels, their differences of climate, are less, than the differences between the upper states of the great valley, and corresponding portions of the Union, near the sea board. First, the elevation of the land above the surface of the ocean, is nearly the same in Louisiana, Arkansaw, Mississippi, and Alabama, as in Georgia and South Carolina.—Second, their geographical relation to the sea, is similar. Third, neither of them is contiguous to mountains, except the N. W. portions of S. Carolina. Hence the emigrant from the banks of the Santee or Savannah river, to those of the lower Mississippi or Red River, if he keep in the same latitude, will experience but little change of climate. He will observe, however, in his new residence, that N. E. winds, attended with cool rains, are less frequent; and on the contrary, that the S. W. wind is more prevalent and perhaps more humid; as it does not, like that of the country he had left, undergo, to any extent, the modifying influence of the peninsula of Florida. The northern parts of Alabama and Mississippi, are occasionally visited with snows several inches deep, and frosts that congeal the surface; but their duration is transient; while in the southern parts of the same states, in Arkansaw and in Louisiana, these phenomena are much rarer. In their upland parts, these warm states have, on the whole, winters dry, frosty, and bracing: in the maritime portions, raw, humid, and rainy. In the former, the summers are fair and not intemperate; in the latter, hot, infested with thunder gusts, and often sultry.

The inhabitant of New England, or, indeed, of any of the old states north of the Potomac, who starts on a migration to the states which are bounded on the South by the Gulph of Mexico, should be prepared to encounter a decided change of climate, consisting, in increased heat throughout the year, and, especially, in a prolonged summer; in more copious rains, and a greater prevalence of S. W. winds.

The emigrant from the shores of the Mediterranean, will find in this portion of the valley of the Mississippi, the nearest approach to his own climate, though eight or ten degrees further south; but he should prepare himself for greater extremes between summer and winter, than he has been accustomed to in Italy or the south of France.

The greater emigration to the upper or temperate Mississippi states, than to the lower, renders the study of their climate compared with the Atlantic states and Europe, more interesting than the inquiry through which we have just passed.

As in most cases, it is not less useful to inquire into causes, than to look merely at effects, I shall direct the attention of the Profession, and of those who contemplate an emigration from the Atlantic to the Western states, to some of the physical circumstances which may be presumed to modify the climates of the two regions.

First, the Atlantic states have an ocean on one side, lakes on another, and mountains, between 2000 and 3000 feet high, on a third; the first and last of which, are so contiguous to the emigrating portions of these states, as to exert a decided influence on their climate. On the other hand, the upper or temperate Mississippi states, are remote from the sea, and comparatively remote from mountains, but have on one side, more extensive lakes, than those which skirt the Atlantic states. The ocean lies to the *east* of the latter, and the Alleghany Mountains to the *west*, while the states of the interior, have the lakes to the

*north*, and the Alleghanies to the *east;* varieties in the physical geography of the two sections, that cannot fail to exert an influence on the temperature and humidity of their winds. Thus the N. E. and S. E. winds of the sea board, are always much more damp than those of the interior. In spring, summer, and autumn, they bring more copious rains, and in winter, deeper snows. They are, also, more frequent and violent, than in the upper Mississippi states. Finally, in winter they are warm; and in summer, as coming from the surface of the ocean, would be more fresh and temperate, than in the valley of the Mississippi, were it not for the Alleghany Mountains, which cool the currents which roll over them to that region. These mountains on the other hand, reduce the temperature and refresh the S. W. and W. winds, which blow from the interior of the continent towards the Atlantic Ocean, and give to the people of the middle maritime states, a succession of less sultry breezes in summer, than envelope their brethren in the west. For a similar reason, the N. W. wind is drier and colder, at the same season, on the leeward, than the windward side of the Alleghanies.

Second, The courses of rivers, by changing, in some degree the direction of the winds, exert an influence on our climates. In the Atlantic states, from New England to North Carolina, the rivers run more or less to the S. E. and deflect the winds which blow from the West; while the great bed of the Mississippi, exerts an equal influence in augmenting the number and steadiness of the winds, which blow over it from the S. W.; and here is another cause of difference in climate, chiefly perceptible, first, in the temperature, which, if no counteracting cause existed, they would raise in the West, considerably above that of corresponding latitudes in the East; secondly, in the moisture of the two regions, which is generally greater, west than east of the mountains, when the S. W. wind prevails, as much of the water with which it comes charged from the Gulph of Mexico, is deposited before it reaches the country east of the Alleghanies.

Third. The great cities of Boston, New York, Philadelphia and Baltimore, are but a few feet above the level of the sea, and the country connected with them has but little elevation; while the towns which are rising on the banks of the upper Mississippi, the Missouri, Wabash, Ohio, Cumberland and Tennessee rivers, are elevated about 550 feet above the surface of the ocean, and the intervening country, about 250 feet more. The influence of this elevation, so often overlooked in comparing the climate of the Eastern and the Western states, would give cooler summers to the latter than the former, were it not for the greater prevalence of S. W. winds in the region of greater elevation. To estimate the full effect of this superior elevation, it is necessary to refer to the increasing altitude, of the immense, unwooded plain, down which the Missouri flows, from the Chippewan or Rocky Mountains, which themselves rise to the varying height of 5, 6, or 7000 feet, and give to the western winds, which would, otherwise, arrive moist and temperate, from the Pacific Ocean, a decided character of transparency and coldness.

Fourth. The general inclination of the surface of the Atlantic states, is to the E. and S. E.—that of the Mississippi states to the S. Were these angles of inclination great, their effects upon the temperature of the two regions would be decisive; but being slight, the increased heat of the West, from this cause, is not, perhaps, very considerable

Fifth. The West abounds in lofty forests of far greater extent than the East; an element of difference in the climates of the two, the inflence of which must beadmitted, although its exact amount cannot, perhaps be estimated. Forests are said to retard the velocity of the winds, and increase the precipitation of their moisture. It is more certain, that in spring, summer, and autumn, they intercept the rays of the sun, most of which are thus reflected before they reach the surface of the earth, and copious evaporation is thus prevented. They seem, also, to promote the condensation of the vapor, from the surrounding air; and may be considered as one of the causse of the more copious dews, which are said to fall west than east of the mountains.

Such are the leading causes, of any differences of climate, which may exist between the states which lie on the sea, and those on the upper Mississippi, and its tributaries. These differences, especially in temperature, have been much dwelt upon, ever since the publication of Mr. Jefferson's celebrated Notes on Virginia, in 1781. But as it respects the heat of summer, they have been greatly overrated. Two causes have contributed to augment and perpetuate the error; first, the extensive popularity of Mr. Jefferson's book; secondly, the great number of emigrants from New England and New York, most of whom have been unconscious how much they had changed their latitude in a migration to the interior, and have made reports concerning the heat of the latter, in the same language as if they had travelled on the latitudes on which they were born. As a general fact, the *settlers* from Virginia in Kentucky, and from Maryland and Pennsylvania, in the states north of the Ohio, have not complained of hotter summers, than they had been accustomed to experience in their native land. It is probable, however, that the summer temperature of the West, *is* rather greater than that of the East, in corresponding latitudes; an effect, as far as it exists, to be ascribed chiefly to the greater prevalence of S. W. winds, in the former, than the latter, and to the influence of the Alleghany Mountains, over which those winds must pass to reach the Atlantic states. Both these causes, however, are in a good degree compensated, by the greater elevation of the interior, as already pointed out. So great is the effect of this elevation, that in the middle of the state of Ohio, which it is nearly 1000 feet above the level of the sea, the temperature is probably lower, than in corresponding latitudes of eastern Pennsylvania.

To solve the problem of comparative temperature, accurate cotemporary observations, should be made on compared thermometers, placed under similar circumstances, in a great number of situations; but this has not yet been done. The only points, that admit of a comparison approaching to this, are Cincinnati and Philadelphia, or its vicinity—the former, however, situated about 50 minutes south of the latter.

From a series of daily observations in Cincinnati or its vicinity, for eight successive years, the mean annual temperature has been ascertained to be 54 degrees and a quarter; Dr. Rush states the mean heat of Philadelphia at 52 degrees and a half, Dr. Coxe, from six years observations, at 54 and a sixth, and Mr. Legaux, from 17 years observations at Springmill, a few miles out of that city, at 53 and a third; the mean term of which results, 53 and a third, is but the fraction of a degree lower, than the mean heat of Cincinnati, and actually less, than should be afforded by the difference of latitude.

A reference to the mean temperatures of summer and winter, will give nearly the same results. From nine year's observations, (three at Springmill by Mr. Legaux, and six in Philadelphia by Dr. Coxe,) the mean summer heat of that part of Pennsylvania, appears to be 76 degrees and six tenths. The mean summer heat at Cincinnati, for an equal number of years, was 74 and four tenths. The average number of days, in which the thermometer rose to 90 degrees or upwards, during the same period, was 14, each summer; and the greatest elevation observed was 98 degrees: all of which would bear an almost exact comparison, with similar observations in Pennsylvania. Mr. Legaux states the most intense cold, at Springmill, from 1787 to 1806, to have been 17 and five tenths degrees below cipher—while within the same period, it was 18, at Cincinnati. The average of extreme cold for several years, as observed by Mr. Legaux, was one and eight tenths of a degree below cipher: the same average at Cincinnati, is two degrees below. From all which we may conclude, that the banks of the Delaware and Ohio, in the same latitudes, have nearly the same temperature.

Let us recur to the winds, the great modifiers of climate in every country. I have already referred to the greater prevalence of S. W. winds in the interior—of N. E. and N. W. winds in the maritime states. Not having at hand the materials for an extended comparison, I shall insert a tabular view of observations made at Cincinnati, for six succeeding years, with so few omissions, that they amount to 4209. They have been brought to the eight principal points of the compass; and while they show the relative prevalence of different winds in the successive months of the year, will furnish data for comparisons between the Western and Eastern states, by those who reside and observe in the latter.

OBSERVATIONS.

| | S. E. | S. | S. W. | N. E. | N. | N. W. | E. | W. | Calm. |
|---|---|---|---|---|---|---|---|---|---|
| January | 6 | 2 | 13 | 8 | 1 | 21 | 3 | 6 | 6 |
| February | 5 | 1 | 13 | 8 | 1 | 14 | 0 | 5 | 8 |
| March | 10 | 1 | 16 | 11 | 1 | 10 | 0 | 5 | 4 |
| April | 7 | 0 | 24 | 10 | 1 | 8 | 1 | 3 | 5 |
| May | 7 | 1 | 19 | 10 | 0 | 10 | 1 | 4 | 6 |
| June | 9 | 1 | 23 | 12 | 5 | 7 | 1 | 2 | 3 |
| July | 6 | 1 | 19 | 11 | 2 | 11 | 1 | 4 | 4 |
| August | 6 | 1 | 23 | 10 | 1 | 12 | 1 | 1 | 6 |
| September | 6 | 1 | 23 | 9 | 0 | 8 | 2 | 3 | 3 |
| October | 9 | 1 | 24 | 6 | 1 | 10 | 2 | 4 | 3 |
| November | 9 | 3 | 13 | 6 | 1 | 10 | 2 | 7 | 5 |
| December | 7 | 1 | 11 | 5 | 0 | 15 | 2 | 6 | 9 |
| | 87 | 14 | 221 | 106 | 14 | 136 | 16 | 50 | 62 |

From this table it appears, 1. That the different winds of Cincinnati prevail in the following order: south-west—north-west—north-east—south-east—west-east—south and north. 2. That the south-west is the prevalent wind, nine months out of the twelve, viz: from March to November, inclusively. 3. That the N. W. wind prevails in January, December, and February. 4. That the greatest number of calm days are in December and February; the least in June, September, and October; which are equal. 5. That the southern are to the northern winds, as 322 to 256; or about 40 to 32. 6. That the western are the prevalent winds throughout the whole year, being to the eastern as 407 to 200, or merely 4 to 2. 7. That the west wind blows only half as much in the six warmer, as in the six colder months. 8. That the east, south, and north winds are nearly equal.

Most of these deductions are exhibited by the following table, in which the whole number of observations, stated above, are supposed to be represented by 1000, and the subsequent numbers to be its fractional parts.

Mean of 6 year's observations—1000, of which the

| | | |
|---|---|---|
| South-east make | - - | 122 |
| South, | - - - | 19 |
| South-west, | - - - | 313 |
| Southern, | - - - | 454 |
| North-west, | - - - | 192 |
| North, | - - - | 19 |
| North-east, | - - - | 150 |
| Northern, | - - - | 361 |
| East, | - - - | 22 |
| Eastern, | - - - | 294 |
| West, | - - - | 70 |
| Western, | - - - | 575 |
| Calm, | - - - | 87 |

A brief sketch of the character of the principal winds embraced in the tables may be useful to those who purpose to emigrate to the West.

1. *The South-West.* This wind, which, as we have just seen, prevails on the Ohio three fourths of the year, exhibits two different characters, or is divisible into two varieties—the *humid* and the *arid.* The former of these is characterized by prevailing throughout the night; by generally continuing two or three days after its commencement; by alternating with the north-east wind, by sinking the barometer more than any other ærial current; and by always causing clouds and generally rain, which is often profuse. The arid south-west commences between sunrise and 10 o'clock in the morning. It is at first very gentle, and increases in force with the progress of the day, until 4 or 5 o'clock in the evening, when it begins to subside. About sunset it ceases, and the succeeding night is clear and serene. This is the predominant wind in the hottest and dryest weather, with which indeed it is identified in the mind of every observer in the west. Its prevalence, in comparison with the other variety, is, perhaps, as eight or ten to one. It is seldom attended with an atmosphere altogether cloudless, but never produces any other form of rain than a thunder-shower. It sinks the barometer less than the humid south-west, but raises the thermometer higher than any other wind. It is not known whether at present it prevails more or less than at the first settlement of the western states.

2. *The North-west.* This wind, like that already described, exhibits two varieties, one of which occurs in warm, the other in cool weather. A state of calmness or the dry south-west, generally precedes and follows the former of these varieties. It is the gale which attends thunder-storms, and of course commences to the windward. Its duration is transient, seldom continuing longer than a few hours; and its geographical extent is equally limited. The other, which is the principal variety of north-west wind, begins, it is well known to the leeward; it generally succeeds rain, and may be regarded as the harbinger of fair weather. In spring and autumn, however, it is frequently attended with moderate showers, which seldom continue more than a day; and in winter it produces snows, that are sometimes among the deepest that fall in the valley of the Mississippi.

In common, it does not exhibit any nocturnal intermission, though for the most part it blows with less violence at night than in the day. It is generally followed by a calm, which is succeeded by the south-east or south-west wind. It frequently undergoes a change into the north-east, blowing from every intermediate point of the compass. On the barometer and thermometer it produces effects opposite to those of the south-west wind. The greatest elevation of the former, and depression of the latter of these instruments, hitherto observed at this place, were during the prevalence of this wind. The longer it continues, the lower is its temperature; and when it is not too much reduced, it feels as pleasant as it is uniformly pure and invigorating.

3. The North-East. This current, by ascending the St. Lawrence, *may* reach Cincinnati without passing over the Alleghanies; but it generally traverses those mountains, and deposits on them, as already stated, a part of its humidity, as appears from its seldom producing much rain or snow along the Ohio. Except, however, when it succeeds to the moist south-west, and follows a storm, this wind constantly produces one of those, or at least cloudy weather. In temperature and weight, it holds a medium between south-west and north-west. It sometimes continues to blow for a week after a south-west storm, during which the sky will perhaps be nearly clear. It is invariably moist, and produces in all exposed to it, the sensation termed *rawness;* though in a much less degree than in the Atlantic states.

4. The South-east. This partakes much of the character of the humid south-west, for it raises the thermometer and sinks the barometer in a moderate degree. It is always damp, and generally produces rain or snow. It frequently succeeds to the north-west, and is then for the most part attended with a clear sky.

5. *The West.* This is generally a cool and rapid wind. From the region it traverses in reaching this place, it must necessarily be dry and enlivening. In the winter, when it continues long enough for the air of the Chippewan Mountains to arrive, it produces intense cold, sinking the thermometer sometimes below cipher.

6. *The North-east and South.* These winds do not prevail respectively more than one week in the year. The first seems to possess most of the qualities of the north-west, and the second of the north-east; the third appears to be a modification of the humid south-west, and is always stormy.

In regard to the weather, on the Ohio, the following table, setting forth the results of 4,268 observations, will afford sufficient information for the general reader. Observations made in other parts of the upper Mississippi states, would probably, give nearly the same proportions of clear and cloudy weather.

| *Clear days.* | *Cloudy days.* | *Variable days.* |
|---|---|---|
| 1—180 | 107 | 68 |
| 2—158 | 112 | 91 |
| 3—187 | 78 | 85 |
| 4—152 | 106 | 107 |
| 5—185 | 111 | 68 |
| 6—172 | 112 | 74 |
| Mean terms 172. 33 | 104. 33 | 82. 16. |

From these results it may be expected that, of the 365 days in the year, about 176 will be fair, 105 cloudy, and 84 variable.

The condition of the weather, in each month of a mean year, for the above period, is exhibited in the following statement.

| | Clear days. | Cloudy days. | Variable days. |
|---|---|---|---|
| January, | 9. 8 | 13. 1 | 7. 8 |
| February, | 10. 3 | 12. 0 | 6. 5 |
| March, | 13. 5 | 9. 1 | 8. 3 |
| April, | 13. 1 | 10. 8 | 7. 6 |
| May, | 15. 0 | 8. 5 | 7. 5 |
| June, | 15. 5 | 5. 0 | 9. 6 |
| July, | 19. 0 | 5. 5 | 6. 0 |
| August, | 19. 6 | 4. 6 | 6. 5 |
| September, | 19. 5 | 5. 3 | 6. 1 |
| October, | 16. 1 | 6. 0 | 8. 1 |
| November, | 9. 5 | 13. 5 | 5. 5 |
| December, | 9. 6 | 14. 1 | 5. 8 |

From this table it appears, that July, August, and September have the greatest, and about an equal number of fair days; that October, June, and May, compose the next class; to which succeed the months of March and April, followed by February, December, January, and November; that in the four latter months there is the greatest proportion of cloudy weather; that next to these rank April, March, and May, succeeded by the remaining months, which are nearly equal. Lastly, that in the number of days which are variable, according to the sense in which that term is here applied, there is among the months no great difference.

The quantity of water in the form of rain and snow, which falls annually in the west, has not been accurately ascertained. It is probably about 36 inches, and nearly the same as in corresponding latitudes, east of the mountains. Taking the mean of a series of years, it is found that in April and May there falls the largest quantity; next to these are November, March, December, July, and October, succeeded by January, August, February, September and June. The same month in different years, affords very different quantities of rain. September has been observed to vary in this respect, from less than an inch to more than five; October from half an inch to eight; and April from two to nine. The spring rains are sometimes excessive, and protracted for eight or ten weeks, during which there are showers, perhaps on an average every third day. During the spring of 1813, there fell upwards of sixteen inches; four times the quantity which fell in the ensuing four months. At other times this state of things is reversed. In the spring of 1814, there fell not more than nine inches; and in the three subsequent months the quantity was equal to fourteen.

Every irregular distribution of the spring and summer rains is of course prejudicial to agriculture. The copious and long continued storms of the former season, now and then check the early growth, or even prevent the planting of many important vegetables. To these rains such dry summers occasionally succeed, that the pastures are consumed, the leaves of the Indian corn become curled, and those of many forest trees, in dry situations, die and fall off before the usual time. But, fortunately, such extraordinary droughts occur too seldom, and are too limited in their extent, to be regarded as any great calamity.

From the valley of the Tennessee river, to the summit level, between the Lakes and the waters which flow into the Ohio, the snows become deeper and deeper. In Tennessee they seldom exceed a few inches—in the centre and north parts of Ohio and Indiana, they sometimes, as was the case in 1830—1, fall to the depth of two feet. North of the Ohio river, the snows are deeper in proportion to the latitude, than they are on the opposite side; which is owing, perhaps, to two causes; first, the greater elevation of the interior of Illinois, Indiana, and Ohio; secondly, the vicinity of the lakes. At Cincinnati, the deepest snow which falls, does not exceed a foot; but one third of that depth is a more common maximum for the winter. This being the case, and periods of mild weather with rain occurring frequently, in almost every winter month, the ground is seldom covered for any great length of time. Severe winters, however, have occurred a number of times, in which the same snow has continued on the ground for several weeks, during which the Ohio was bridged with ice from its sources to its mouth. This happened as far back as 1796, and was repeated as late as 1831—2, showing that no particular change of climate has yet taken place in the west.

More snow probably falls east of the mountains than west, below the latitude of 40 deg.; above the parallel, the difference is not perceptible. This can be easily explained. In the maritime states every eastern wind, in winter, almost of necessity brings snow, as it transfers the atmosphere of the sea, over the colder surface of the continent. In the west, the great lakes supply, in this respect, the place of the Atlantic Ocean, and augment the depth of the snow for two degrees south of their shores. The fogs of the western rivers are said to be denser, than those of the eastern, but additional facts are necessary to an accurate estimate.

In tornadoes, hailstorms, winter thaws, and floods, summer frosts, premature springs, anticipating autumns, and other anomalies, the climates of the east and west, in the same latitudes, seem not materially to differ.

In the interior states, the pleasantest travelling and emigrating months, are April, May, and June; September after the equinox, the entire month of October, and the first half of November. The vernal travelling season, is more showery than the autumnal, but from the fulness of the rivers, and the lively green of the forests and fields, more cheerful. The autumn is smoky, dusty, and often deficient in water, but serene, equable in temperature, and decorated with leaves of every tint, which present the traveller of taste with an untiring succession of picturesque and beautiful views.

June, 1832.

